# Heparan Sulfate and Heparan Sulfate Proteoglycans in Cancer Initiation and Progression

**DOI:** 10.3389/fendo.2018.00483

**Published:** 2018-08-24

**Authors:** Arvindhan Nagarajan, Parmanand Malvi, Narendra Wajapeyee

**Affiliations:** ^1^Department of Pathology, Yale University School of Medicine, New Haven, CT, United States; ^2^Yale Cancer Center, Yale University School of Medicine, New Haven, CT, United States

**Keywords:** heparan sulfate, heparan sulfate proteoglycans, cancer, immune evasion, signaling

## Abstract

Heparan sulfate (HS) are complex unbranched carbohydrate chains that are heavily modified by sulfate and exist either conjugated to proteins or as free, unconjugated chains. Proteins with covalently bound Heparan sulfate chains are termed Heparan Sulfate Proteoglycans (HSPGs). Both HS and HSPGs bind to various growth factors and act as co-receptors for different cell surface receptors. They also modulate the dynamics and kinetics of various ligand-receptor interactions, which in turn can influence the duration and potency of the signaling. HS and HSPGs have also been shown to exert a structural role as a component of the extracellular matrix, thereby altering processes such as cell adhesion, immune cell infiltration and angiogenesis. Previous studies have shown that HS are deregulated in a variety of solid tumors and hematological malignancies and regulate key aspects of cancer initiation and progression. HS deregulation in cancer can occur as a result of changes in the level of HSPGs or due to changes in the levels of HS biosynthesis and remodeling enzymes. Here, we describe the major cell-autonomous (proliferation, apoptosis/senescence and differentiation) and cell-non-autonomous (angiogenesis, immune evasion, and matrix remodeling) roles of HS and HSPGs in cancer. Finally, we discuss therapeutic opportunities for targeting deregulated HS biosynthesis and HSPGs as a strategy for cancer treatment.

## Introduction

Normal cells acquire series of genetic and epigenetic aberrations to become cancerous. The acquired cancer growth and progression enabling attributes are collectively referred to as hallmarks of cancer (1). Several hallmarks of cancer, such as sustained growth signaling, suppression of apoptosis, deregulated metabolism, immune evasion and angiogenesis can also be enhanced through pathological alterations of normal physiological processes (1).

Heparan sulfates (HS) are unbranched chains of disaccharide repeats that are heavily sulfated at various positions on their sugar residues (2, 3). HS can occur either conjugated to amino acids, creating heparan sulfate proteoglycans (HSPGs), or as unconjugated chains (4). Both HS and HSPGs play important roles in cancer initiation and progression. Previous studies have implicated the role of HS and HSPGs in several types of solid tumors as well as hematological malignancies (5–11).

**Figure 1:**
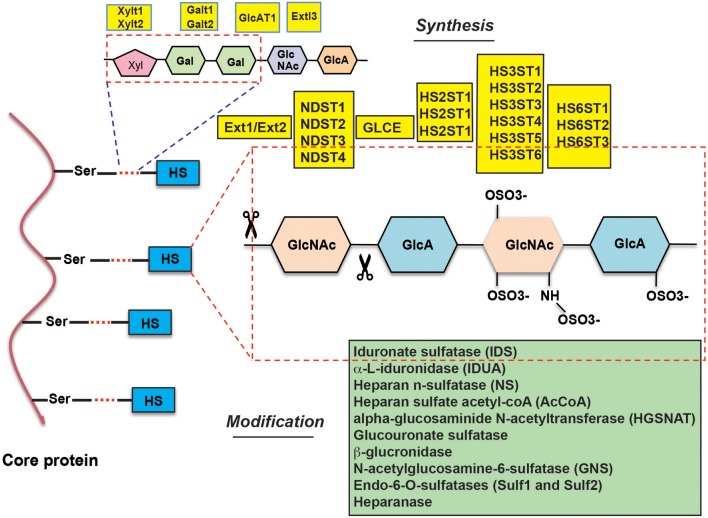
Overview of the enzymes involved in heparan sulfate synthesis and modification.

HSPGs are complex biopolymers whose synthesis is orchestrated by many enzymes, which catalyze the various steps of HS synthesis with very little redundancy (Figure 1). The majority of HS deregulation in cancer occurs due to alterations in the expression of HS-synthesizing and HS-modifying enzymes, however, alterations in HSPGs can also contribute to HS deregulation (12) (also see Table 1) (10, 11, 13–84).

**Table 1:** Deregulation of HS and HSPGs and enzymes involved in HSPG metabolism in cancer.

	**Alteration in cancer**	**Functional consequence(s)**	**Cancer type(s)**
**Enzyme involved in HSPG metabolism**			
HS2ST1	Upregulated	Promote cell proliferation, invasion and growth factor signaling	Prostate cancer (13)
HS3ST2	Epigenetic silencing	Suppression of tumor growth and invasion	Lung cancer (14)
HS3ST2	Upregulated	Invasion and migration	Breast cancer (15)
HS3ST3B1 and HS3ST4	Upregulated	Promote cell proliferation, invasiveness, and tumor angiogenesis	acute myeloid leukemia (16) Colorectal cancer (17) Pancreatic cancer (18)
HS6ST1 and HS6ST2	Upregulated	Increased tumor Angiogenesis	Ovarian cancer (19)
HS6ST2	Upregulated	Poor survival of patients	Colorectal cancer (20)
HS6ST2	Upregulated	Bone metastasis	Breast cancer (21)
HPSE	Upregulated	Tumor metastasis and angiogenesis	Neuroblastoma (22), breast cancer (23), prostate cancer (24), colon cancer (25), lung cancer (26), liver cancer (27), ovarian cancer (28), and pancreatic cancer (29), human myeloma (30)
NDST1 and NDST2	Upregulated	Tumor progression	Hepatocellular carcinoma (31)
SULF1	Downregulated	Suppress tumor cell proliferation and invasion	Breast cancer, Pancreatic, Ovarian and head and neck cancers (32) Hepatocellular carcinoma (33)
SULF2	Unaltered	Tumor progression	Hepatocellular carcinoma and glioblastoma (34)
SULF2	Upregulated	Tumor growth	Hepatocellular carcinoma (33, 35, 36)
**HSPG**			
Agrin	Elevated	Angiogenesis	Hepatocellular carcinoma (37, 38), glioblastoma (39), cholangiocarcinoma (37)
CD44	Elevated	Adhesion, invasion, cancer stem cell	Breast cancer (40), colorectal cancer (41), oral squamous cell carcinoma (42), melanoma (43) Neuroblastoma (44)
Collagen XVIII	Reduced	Angiogenesis	Cutaneous squamous cell carcinoma (45, 46)
GPC1	Elevated	Proliferation	Breast cancer (47), pancreatic ductal adenocarcinoma (48), glioma (49)
GPC3	Elevated	Proliferation	Hepatocellular carcinoma (50), follicular thyroid cancer (51), testicular germ cell tumor, neuroblastoma (52), Wilms' tumor (53), yolk sac tumor (54), lung squamous cell carcinoma (55), hepatoblastoma (56)
GPC5	Elevated	Proliferation, invasion	Rhabdomyosarcoma (10), non-small cell lung cancer (57)
	Reduced	Initiation	Non-small cell lung cancer (58)
Perlecan	Elevated	Proliferation, angiogenesis	Prostate cancer (59), hepatoblastoma (60), pancreatic ductal adenocarcinoma (61), melanoma (62)
SDC1	Elevated	Proliferation	Breast cancer (63), pancreatic ductal adenocarcinoma (64), ovarian cancer (65), multiple myelom (66)
SDC2	Elevated	Adhesion, proliferation	Breast cancer (67), prostate cancer (68), colorectal cancer (69), bladder cancer (70), glioma (71), sarcoma (72)
SDC3	Elevated	Perineural invasion and poor prognosis	Pancreatic ductal adenocarcinoma (73)
SDC4	Reduced	Differentiation	Neuroblastoma (11)
TbRIII	Elevated	Migration, proliferation	Colon cancer (74), non-Hodgkin's lymphoma (75),
	Reduced	Invasion, proliferation, differentiation, immune response	Breast cancer (76), prostate cancer (77), ovarian cancer (78), multiple myeloma (79), neuroblastoma (11), non-small cell lung cancer (80), pancreatic ductal adenocarcinoma (81), endometrial cancer, renal cell carcinoma (82), melanoma (83)

In this review, we provide an overview of the cell-autonomous and cell-non-autonomous roles of HS and HSPGs in cancer initiation and progression. In addition, we will also discuss opportunities to develop cancer therapies by targeting the HS and HSPG axis.

## Cell-autonomous role of HS and HSPGs in cancer initiation and progression

HS and HSPGs regulate diverse cell-autonomous functions, including oncogenic signaling, apoptosis, and cellular differentiation. In this section, we describe the cell-autonomous functions of HS and HSPGs in cancer initiation and progression.

### Growth factor signaling and regulation of proliferation

Previous studies have shown important roles of HS and HSPGs in oncogenic signaling (85–88). In this regard, FGF binding interactions are best characterized by the role of HS in altered Receptor Tyrosine Kinase (RTK) signaling. For example, HS-modified HSPGs bind FGF ligands and receptors to form a ternary complex and enhance signaling by promoting FGF receptor (FGFR) dimerization (89–91). This in turn results in receptor activation and enhanced FGFR signaling, which consequentially promotes tumor growth (89–91). In addition to FGF, HS binds to several different mitogenic growth factors such as PDGF, Heparin-Binding Epidermal Growth Factor-like Growth Factor (HB-EGF), and Hepatocyte Growth Factor (HGF) and modulates their signaling in a context dependent manner (86).

Breast cancer cells are also shown to overexpress HSPGs, such as Glypican 1 (GPC1) and Syndecan 1 (SDC1), which enhance the proliferative response after treatment with various growth factors due to prolonged signaling (86). Similar to breast cancer, GPC1 also has been shown to have growth-promoting effects in pancreatic cancer and gliomas (49, 92, 93). Collectively, these studies highlight wide-spread deregulation of HSPGs in different cancers that exert tumor promoting roles.

Additionally, HSPGs also influence cell-matrix interactions by binding matrix proteins such as fibronectin, laminin, thrombospondin, and collagen (89, 94). For example, SDC2 has been shown to be overexpressed in colon cancer cell lines and inhibition of SDC2 in these cells results in cell cycle arrest (69). Similarly, RKIP and HMGB2-dependent breast cancer survival and metastasis was shown to be regulated in SDC2 dependent manner (67). However, it is important to note that in addition to the pro-tumorigenic effects, some HSPGs, such as SDC2, exert tumor suppressive effects depending on the cancer type (95, 96).

Interestingly, HSPGs on the cell surface can also shed, generating soluble proteins that influence cellular proliferation by accumulating in intercellular spaces and sequestering growth factors (89). HSPGs are also often expressed in the tumor stroma and affect several cancer cell growth-enabling features (89). For example, stromal SDC1 that is released into the tumor microenvironment promotes breast carcinoma growth by enhancing FGF2 signaling (97). Interestingly, SDC1 shedding into the stroma is enhanced by heparanase expression, in part through removal and reduction of heparan sulfate chains (30). Thus, various components of the HS signaling pathway coordinate to promote carcinogenesis.

HSPGs secreted into the stroma can also inhibit cancer cell proliferation. For example, increased FGF2 signaling due to soluble HSPGs suppresses neuroblastoma proliferation (11, 98). Specifically, it has been shown that growing neuroblastoma cells with soluble HSPGs promote its differentiation by enhancing both basal and FGF1 mediated phosphorylation of ERK1/2 and expression of transcription factor ID1 (11). Another study has shown that the HSPG, type III TGF-β receptor (TGFBR3) acts as a co-receptor in FGF2 mediated neuroblastome differentiation (98). Similarly, SDC1 that is expressed in multiple myeloma has been shown to activate WNT signaling by two mechanisms (99). First, Wnts bind to the SDC1 HS side chains and activates WNT pathway in a paracrine manner via Frizzled. Second, SDC1 binds to R-spondins produced in osteoblast and stabilizes Frizzled in a LGR4-dependent manner (99). In other instances, soluble HSPGs sequester growth factors, reducing certain pro-proliferative signals. For example, GPC3 promotes hepatocellular carcinoma growth by activating WNT signaling (100). However, contrary to this, soluble GPC3 has been shown to block hepatocellular carcinoma growth by blocking WNT signaling and MAP kinase and AKT pathways (101). Taken together, these studies underpin that HS and HSPGs can exert diverse cancer promoting or inhibitory functions depending upon the context.

### Apoptosis and cellular senescence regulation

HS and HSPGs can also play important role in the regulation of apoptosis and cellular senescence. For example, the upregulation of the RTK signaling pathway by HSPGs induces an anti-apoptotic effect through upregulating phosphatidylinositol 3-kinase (PI3K)- and Mitogen-Activated Protein Kinase (MAPK)-mediated survival pathways (102). Additionally, HS and chondroitin sulfate directly inhibit H_2_O_2_-induced apoptosis by blocking cytochrome c release and caspase-3 and -9 activation (103). Death receptor-mediated apoptosis pathway, which is mediated through the cell surface receptors for Fas ligand (FasL) and Tumor Necrosis Factor-related Apoptosis-Inducing Ligand (TRAIL) can also be regulated by HSPGs. For example, SDC1 suppresses TRAIL-mediated apoptosis in multiple myeloma cells (104). The same study also reported that SDC1 knockdown in lymphoma cells protected them against FasL-mediated apoptosis. In addition to the regulation of apoptosis, a recent study also revealed that heparan sulfation is essential for preventing senescence (105). This study revealed that the depletion of 3'-phosphoadenosine 5′-phosphosulfate synthetase 2 (PAPSS2), an enzyme that synthesizes the sulfur donor PAPS, and the small molecule inhibitor-mediated repression of HS sulfation led to premature cell senescence (105). Collectively, these studies further demonstrate the importance of HS and HSPGs in the regulation of cancer growth relevant cellular processes, such as apoptosis and senescence.

### Cellular differentiation regulation

HS, HSPGs, and HS modifiers have also been shown to determine the cellular differentiation state. In this regard, the role of HS modifiers in regulating epithelial-to-mesenchymal transition (EMT) is noteworthy. EMT plays an important role in metastatic progression and drug resistance (106). Cells overexpressing the HS modifier sulfatase 2 (SULF2) present with reduced levels of the trisulfated disaccharide UA(2S)-GlcNS(6S). This reduction is followed by an increase in EMT markers and WNT signaling (107). Tumor cell-mediated tumor stroma modulation can also suppress differentiation and increase proliferation. The expression of several HSPGs is low in neuroblasts and high in the Schwannian stroma, and neuroblastomas with a high TβRIII, GPC1, and SDC3 expression have improved prognosis (11). The same study also found that soluble HSPGs and heparin promoted differentiation and decreased proliferation through FGFR1 and ERK phosphorylation. Similarly, another study has shown that neuroblastoma differentiation is promoted by release of a GPI-anchored HSPG, Glypican-6 (GPC6) through via Glycerophosphodiesterase (GDE2). This study also found that high GDE2 or low GPC6 level in neuroblastoma predicted significantly increased patient survival (108). These studies are of high significance as they make two major points; first, that the differentiation state of the cancer cells predict survival, and second, that HS and HSPGs are among the key regulators of cancer differentiation states.

## Cell-non-autonomous roles of HS signaling in cancer

Several features of cancer such as sustained angiogenesis, tissue invasion and migration and immune evasion require a complex interplay between more than one cell type and involve multiple organ systems. In this section, we describe the cell-non-autonomous functions of HS and HSPGs in cancer initiation and progression.

### Role in angiogenesis

Angiogenesis is considered a key requirement for cancer growth and progression (109). This is highlighted by the fact that several angiogenesis inhibitors are in clinical trials for cancer treatment (110). HS and HSPGs modify angiogenesis due to their effect on angiogenic factors, such as FGF, PDGF, and VEGF. For exmaple, SDC1 binds to VEGF, and SDC1 shedding increases the VEGF concentration in the matrix and promotes angiogenesis in myeloma (111). The same study also showed that heparanase expression increases SDC1 shedding (112). SDC1 is overexpressed in endothelial cells derived from patients with multiple myeloma. In addition to suppressing cell proliferation, RNAi silencing of SDC1 in patient-derived endothelial cells reduces capillary-like structure organization, which is correlated with reduced VEGF receptor (VEGFR)-2 surface expression (111). Other members of the syndecan family, such as SDC2 and SDC3, also affect tumor angiogenesis (113, 114).

Another HSPG with an opposing effect on angiogenesis is Perlecan. Perlecan is a secreted HSPG which is also found on cancer cell surface and in cancer microenvironment (115). Perlecan is shown to promotes angiogenesis in its intact form (115). However, Perlecan can also be partially cleaved by proteases, which results in a C-terminal fragment, called endorepellin, which has been shown to exert anti-angiogenic effects (116). Thus, HSPGs modulate tumor angiogenesis in multiple ways: they increase the tumor microenvironment VEGF concentration, affect VEGFR surface localization, and fine-tune interaction of VEGF with its receptor and co-receptor.

### Role in immune evasion

Immune response is the first line of systemic defense against tumorigenesis (117). Recent success of immunotherapeutic approaches to treat cancer further highlights the importance of immune evasion mechanisms for cancer initiation and progression (118, 119). HSPGs can serve as cancer biomarkers, which can also be used to target antibodies for immunotherapies (120, 121). At the same time, evidence suggests that HSPGs in the extracellular matrix (ECM) or those expressed on bystander cells are involved in reducing immune signaling to dendritic cells (DCs) (122). One of the well-studied HSPGs roles in melanoma immunity involves myeloid-derived suppressor cells (MDSCs) that suppresses immunity against melanoma (122). Previous studies have shown that melanoma immune evasion involves myeloid-derived suppressor cells (MDSCs) that express an immune-suppressive molecule called dendritic cell-associated, HSPG-dependent integrin ligand (DC-HIL) (122). DL-HIL engages Syndecan-4 on effector T cell causing anergy (122). Furthermore, targeting DC-HIL with neutralizing antibody or its genetic knockout delayed the growth of transplantable B16 melanoma in syngeneic mice, which further strengthen the role of DC-HIL as a potential target for enhancing the immune response and cause tumor eradication (123).

HSPGs also affect innate immune response against cancer cells by modulating Natural Killer (NK) cell-mediated activity against cancer cells. NK cells exert their cytotoxic activity on cancer cells through recognition of specific ligands, one group of which is called the natural cytotoxicity receptors (NCR) (124). The NCRs bind to HSPGs and their interaction promotes NK cell-mediated cancer cell eradication (125). Additionally, it has been shown that cancer cells upregulate heparanase through activation of bromodomain PHD finger transcription factor (BPTF), leading to reduced NCR-HSPG interaction, which results in dampened NK cell response (126). Collectively, these studies demonstrate that by activating immune tolerance, enhancing signaling pathways, and interfering with immune cell-tumor interactions, HSPGs regulate immune evasion functions in cancer cells.

### Role in the regulation of extracellular matrix modification

HSPGs, free HS chains and heparin are structural components of extracellular matrix (ECM) (12). The ECM is a major part of the tumor microenvironment and influences tumor progression by several mechanisms, including growth factor concentrations, angiogenesis, and immune infiltration (127). The changes in HSPGs and HS metabolizing enzymes vary widely with cancer type and have varying context dependent roles.

Right-sided colorectal cancers show that the expression of the HSPGs glypican-1,-3, and -6 and betaglycan are altered in non-metastatic tumors, whereas in metastatic tumors, only glypican-1 and SDC1 are modified. Interestingly, alterations were found in only non-metastatic tumors, affecting N-sulfation, and the isoforms of heparan sulfate 6-O-sulfotransferase 1 (HS6ST1), heparan sulfate-glucosamine 3-sulfotransferase 3B1 (HS3ST3B1) and heparan sulfate-glucosamine 3-sulfotransferase 5 (HS3ST5) (128). The HSPG SDC2 induces MMP-7-mediated E-cadherin shedding in colorectal cancer. E-Cadherin shedding led to reduced cell-to-cell contacts and the acquisition of a fibroblast-like morphology, which are both associated with cancer metastasis (129). Another important study showed that SDC1-positive human mammary fibroblasts (HMF) induced extracellular matrix remodeling by promoting an aligned fiber architecture, which promoted directional migration and invasion of breast cancer cells (130).

Apart from syndecans, perlecan and agrin, two other basement membrane constituents are also involved in cancer progression (131–133). Antisense RNA against perlecan inhibits tumor growth and angiogenesis in colon carcinoma (134). Moreover, the ECM protein agrin stimulated osteosarcoma cell growth and migration. Agrin also induces a switch from topoisomerase I to topoisomerase II (135). Therefore, these studies collectively reveal the role of HSPG ECM constituents and cell surface HSPGs in regulating cell-to-cell and cell-matrix adhesion, which in turn control tumor cell migration and shedding.

## Targeting HS and HSPGs for cancer treatment

Understanding the biology behind HS and HSPG deregulation in cancers has enabled the development of various therapeutic strategies aimed at various HS- and HSPG-mediated cancer growth and progression enabling features. Small molecule inhibitors, which interfere with the activities of various enzymes involved in HSPG synthesis and modification, have been developed (6). Additionally, small molecule inhibitors and monoclonal antibodies, which target interactions between HSPGs and their targets, are being developed (136, 137). Below, we describe some of these agents and their value as anti-cancer agents.

### Antibody and small molecule targeting HS-modifying enzymes, HS, and HSPGs

Among the enzymes involved in HS synthesis and modifications, heparanases, and sulfatases are considered good drug targets. Heparanase is overexpressed in a wide-variety of solid tumors and hematological malignancies (29). A previous study assessed the therapeutic value of heparanase targeting using heparanse-neutralizing antibodies for the treatment of diffuse non-Hodgkin's B-cell lymphoma and follicular lymphoma (138). This study found that heparanase inhibition blocked xenograft tumors and growth of lymphoma cells in the bones of mice (138). Additional studies have shown that antibody-mediated anti-heparanase-therapies inhibit cell invasion and tumor metastasis (138–140). Recently, a small molecule inhibitor of hepranase was developed and was shown to reduce metastatic attributes in a model of hepatocellular carcinoma (141). Thus, these studies collectively establish heparanase as a potential drug target for cancer therapy.

Small molecule inhibitors, which prevent growth hormone binding to HSPG, reduce the proliferative HSPG-mediated signal. A similarity-based screening of small molecule libraries identified bi-naphthalenic compounds, which can inhibit FGF binding to both, HSPGs and FGFR1 binding. *In vitro* and *ex vivo*, these compounds inhibit FGF2 activity in angiogenesis models, with improved therapeutic potency (142). Monoclonal antibodies developed against the HS chain on GPC3 inhibit Wnt3a/β-catenin activation, recapitulating GPC3 knockdown by reducing HCC migration and motility (137).

Small molecule inhibitors against sulfatases have shown promise in inhibiting tumor growth. A disulfonyl derivative of phenyl–tert–butyl nitrone (PBN) called OKN-007 inhibited Sulf2 activity in hepatocellular carcinoma (HCC) cell lines and blocked HCC tumor xenograft growth in mice (136).

HS signaling modulation also affects immune cell trafficking and associated immune responses. Deletion of the glycosyltransferase gene exostosin glycosyltransferase 1 (Ext1), which is essential for HS chain formation, in myxovirus resistance-1 (Mx-1)-expressing bone marrow stromal cells increased hematopoietic stem cells (HSCs) efflux from the bone marrow to the spleen in response to granulocyte colony-stimulating factor. Thus, a therapeutic that targets Ext1 may help mobilize immune cells to target cancer cells (143). For detailed review on the role of different enzymes in HS synthesis and modification readers are referred to a review by Bishop et al. (12).

### Heparan sulfate mimetics

HS mimetics were also used as anti-cancer agents. HS mimetics induce an immune response against lymphoma through activation of natural killer (NK) cells (144). The HS mimetic PG545, in addition to its anti-heparanase and anti-angiogenic effect shows pleiotropic effect by enhancing toll-like receptor 9 (TLR9) activation through increasing the TLR9 ligand CpG in DCs. It was shown that treatment with PG545 resulted in the accumulation of CpG in the lysosomal compartment of DCs. This in turn enhanced the IL-12 production, which was essential for the ability of PG545 to activate NK cells (144). Furthermore, PG545 was also shown to directly bind to WNT3A and WNT7A and inhibits WNT/β-catenin signaling, inhibiting proliferation in pancreatic tumor cell lines (145). These studies further highlight the possibility of using heparin sulfate mimetics as agents for cancer therapy.

### HSPGs as immunotherapeutic targets

Some recent studies have also indicated that the upregulation of HSPGs on cancer cells can be used as unique biomarkers that can be targeted to selectively deliver cytotoxic drugs (146, 147). A recent study that analyzed differential expression of cell surface proteins on neuroblastoma identified the HSPG, Glypican-2 (GPC2) as selectively expressed on neuroblastoma where it enhances neuroblastoma proliferation (148). The researchers were able to develop an antibody drug conjugate that selectively eradicated GPC2 positive neuroblastoma (148). This is another exciting area of emerging research where HSPGs can be exploited to serve as targets for selective drug delivery to cancer cells.

## Conclusion

Recent cancer therapies have largely focused on targeting driver mutations and their downstream effectors. However, the emerging body of evidence now shows that driver-mutations are, in fact, enhanced and modified by a host of other modifications as cancer evolves. HS and HSPG deregulation are major contributing factors to cancer evolution. This review has covered some of the well-established and emerging roles of HS and HSPGs in cancer. However, new, non-canonical functions of HSPGs are still being discovered. For instance, in addition to modulating growth factors and RTK interactions, HSPGs also transport growth factors directly to the nucleus, where these factors modify gene regulation (149). HSPGs have also been shown to influence cancer exosome shedding and uptake, thereby modulating cell-to-cell communication between cancer and healthy fibroblasts, immune cells, and endothelial cells (150, 151). HSPGs can also influence actin cytoskeleton remodeling and cancer cell motility (95). The HSPG, SDC2 binds Ezrin, a cytoskeletal protein (152) and serves as adapter molecules for IGF1 mediated activation of ERK (95). Additionally, HSPGs are implicated in lipoprotein uptake and cellular stress signaling (153, 154). As more researchers validate these findings, newer areas of HS- and HSPG-mediated regulation will be discovered. Additionally, as cancer treatment moves from single target to combination therapies, HS- and HSPG-targeting therapies will likely emerge as a major new direction for cancer therapeutics.

## Author contributions

All authors listed have made a substantial, direct and intellectual contribution to the work, and approved it for publication.

### Conflict of interest statement

The authors declare that the research was conducted in the absence of any commercial or financial relationships that could be construed as a potential conflict of interest.
